# Creativity in the Predominantly Inattentive and Combined Presentations of ADHD in Adults

**DOI:** 10.1177/10870547211060547

**Published:** 2021-12-12

**Authors:** Olivier Girard-Joyal, Bruno Gauthier

**Affiliations:** 1 University of Montreal, Laval, QC, Canada

**Keywords:** creativity, ADHD-I, ADHD-C, divergent thinking, executive inhibition

## Abstract

**Objective:** ADHD and its associated inhibition deficits might promote creativity. However, results in the literature are conflicting, possibly due to the heterogeneity of ADHD. To control for this heterogeneity, creativity, and inhibition were investigated in the predominantly inattentive (ADHD-I) and combined (ADHD-C) presentations. **Method:** Participants were males/females aged 18 to 51, diagnosed with ADHD-I (*n* = 21), ADHD-C (*n* = 19), or without ADHD (*n* = 43). Self-rated Kaufman Domains of Creativity Scale and evaluator-rated figural Torrance Test of Creative Thinking (TTCT) were used for measuring creativity, Stroop task for inhibition, and Conners’ Adult ADHD Rating Scales for ADHD symptoms. **Results:** The ADHD-C group reported higher self-rated creativity than other groups and made more original drawings paired to more abstract titles in the figural TTCT than controls. **Conclusion:** ADHD-C participants were the most creative. This result was more importantly associated with higher degrees of ADHD symptoms rather than poorer inhibition.

## Introduction

ADHD is a neurodevelopmental disorder with a childhood onset affecting 2.58% of the global adult population (Song et al., 2021). It leads to higher rates of failure to graduate high school, a higher likelihood to be unemployed, and makes developing and maintaining relationships difficult (Biederman et al., 2006). On the bright side, authors have wondered if ADHD is also associated with a strength: enhanced creativity (Hoogman et al., 2020).

Although this is disputed (Westmeyer, 1998), most studies that will be cited in the present work refer to creativity as the ability to generate ideas that are both novel and useful (Amabile, 2018; Cohen, 2011). Children with ADHD were initially suspected to possess superior creativity in the 1980s because their features (impulsive, distractible, and disorganized) echoed those of creative individuals (adventurous, nonconforming, and impulsive; Levine & Melmed, 1982; Torrance, 1988). Conversely, creative people present certain characteristics associated with ADHD. In samples of creative children and adolescents, 26% to 40% displayed clinically elevated symptoms of ADHD (Cramond, 1994; Healey & Rucklidge, 2006). Apart from similarities between these two populations, ADHD-like behaviors and symptoms are thought to contribute positively to creativity. For instance, mind wandering would be beneficial to creativity in a problem-solving task by creating opportunities to develop (“incubate”) ideas (Baird et al., 2012). Moreover, an impulsive personality may help to generate more new ideas (Brem & Utikal, 2019) and, in employees, impulsive behaviors could predict creative ideas (Gozukara, 2016).

Taken altogether, these pieces of evidence lead to believe that the ADHD population could be highly creative. To verify this, two particular dimensions of the 4P framework of creativity were investigated: the “Person” (i.e., what defines a creative person in terms of personality traits, temperament, behaviors, etc.) and the “Process” (i.e., the psychological factors involved in creativity; Rhodes, 1961). In other words, investigators were especially interested in verifying whether individuals with ADHD presented as creative-looking beings or if they were prone to creative thinking. The Person dimension of creativity is mostly assessed with self-rated scales measuring achievements and behaviors considered creative (e.g., publishing a book) or the self-perception of creative abilities (Kaufman, 2012; Silvia et al., 2012). The Process dimension of creativity is usually evaluated with time-constrained experiments that aim to measure the psychological operations supporting the creative process. Most tasks are measuring divergent thinking (DT) and/or convergent thinking (CT), two concepts introduced by Joy Paul Guilford in its Structure of Intellect theory (Guilford, 1967). DT is the ability to generate many spontaneous and novel ideas. A DT task may require finding new uses for an object (e.g., a brick can be used as a paperweight). Performance is then rated on at least some of the criteria reflecting the different components of DT: fluency (number of ideas), originality (rarity of the ideas), flexibility (variety of ideas), and elaboration (expansion from an idea). CT stands in opposition to DT. It is often comprehended as the ability to find the most appropriate solution to a problem (Guilford, 1967), but is also coined as the fusion of ideas (Lubart, 2016). In a CT task, examinees may be presented with three words (e.g., right-cat-carbon) and asked to find a fourth one that associates them all (e.g., “copy”; Mednick, 1962), for instance. Both DT and CT can be used alternatively throughout the creative process (Lubart, 2016). As an example, a marketing team could brainstorm about the different ways they can advertise a product before narrowing down and refining their best idea. Likewise, a DT task may partly solicit a meta control state associated with CT and vice-versa (Zhang et al., 2020). Thus, it is more adequate to comprehend creative thinking tasks as tools measuring, in various importance, both DT and CT rather than tasks assessing exclusively either one of these two processes.

A recent literature review by Hoogman et al. (2020) breaks down the findings of 23 behavioral studies on creativity in children and adults with an official diagnosis of ADHD. It reveals that three of the four studies using creativity scales reported higher self-rated creativity in adults with ADHD compared to controls, more precisely in terms of creative achievements. Results from DT tasks studies are more conflicting. Out of 14 clinical case-control studies on ADHD (9 in children, 5 in adults), two studies in children and three in adults found that participants with ADHD performed significantly better, especially in terms of originality. However, out of the five case-control studies presenting results from CT tasks, three within the children population found no differences between groups, and the other two, respectively in children and adults, found worst performances in the ADHD groups. Thus, when ADHD groups outperformed control groups, it tended to be in terms of self-reported creativity and DT rather than CT. This tendency could be shaped by executive inhibition abilities.

Executive inhibition (or cognitive inhibition), as defined by Barkley (2005), is the ability to inhibit a prepotent response and control interference. Fragilized inhibitory processes may hinder performances to CT tasks, which require inhibiting intrusions to stay focused until the right answer is found (Fiore et al., 2001; Howard-Jones & Murray, 2003). Executive inhibition functioning may also have an incidence on DT abilities, but this relation is not as straightforward. Some findings support the idea that better executive inhibition is associated with higher creativity, especially in terms of DT abilities (Benedek et al., 2012, 2014; Edl et al., 2014). Executive inhibition would help suppress prepotent ideas in favor of original ideas. Interestingly, other findings show that poorer executive inhibition may also be advantageous to DT. In adults without ADHD, poorer performance to a reading inhibition task is associated with higher idea generation to a DT task (Fiore et al., 2001). Carson et al. (2003) also discovered that lower latent inhibition could contribute to DT by facilitating associations between ideas. Latent inhibition is a concept closely related to interference control in executive inhibition. It refers to the capacity to filter out familiar stimuli from the environment in profit of new stimuli (Lubow, 1989). Lower latent inhibition is also associated with more creative achievements (Carson et al., 2003).

The case-control study in adults with ADHD of White and Shah (2006), which is part of the systematic review of Hoogman et al. (2020), revealed that participants with ADHD performed worst to a CT task and better to a DT task than participants without ADHD. Executive inhibition, sometimes impaired in ADHD (Nigg, 2001), was measured by a proactive interference task and appeared to mediate CT, but not DT. As the authors explained, executive inhibition is not a unitary concept and ADHD is associated with multiple inhibitory deficits, so other ADHD-related inhibitory deficits might better contribute to DT. For instance, the implication of response inhibition on creativity (Barkley, 1999) has yet to be investigated within this population. Thus, particularities related to executive inhibition may explain some of the variability of the results between CT, DT, and creative behaviors within the ADHD population. Still, judging by the review of Hoogman et al. (2020), there also appears to be considerable variability between the behavioral studies that used the same type of tasks. This may be attributable to the heterogeneity of the ADHD samples.

The most prevalent presentations of ADHD are combined (ADHD-C) and predominantly inattentive (ADHD-I; American Psychiatric Association (APA, 2013). However, previous work on creativity overlooked this subdivision of the disorder or only considered the combined presentation. This is an issue since reviews have shown that individuals with ADHD-I and ADHD-C may perform differently to standard measures of inhibition like the cued Go/No-go task (Adams et al., 2008) and the Stroop task (van Mourik et al., 2005), suggesting the existence of specific response inhibition alterations for these two presentations of ADHD. Besides, only a portion of those who suffer from ADHD present significant impairments (Nigg et al., 2005). Considering the shreds of evidence supporting a hypothetical role of executive inhibition functioning in creativity, variations of inhibition fragilities between and within presentations of ADHD may contribute to the variability of the results observed by Hoogman et al. (2020).

As just mentioned, ADHD is a heterogeneous disorder in terms of behavioral symptoms (Wåhlstedt et al., 2009) andcertain seem to be more beneficial to creativity than others. In children, teacher-rated impulsive/hyperactive symptoms were related to higher fluency but low inattention was related to better flexibility (Brandau et al., 2007). In adults, self-reported ADHD symptoms (especially impulsivity and hyperactivity) were positively correlated to DT and the number of creative achievements and behaviors in self-rated questionnaires (Boot et al., 2017b). Thus, impulsivity and hyperactivity seem to be the main drivers of superior self-rated creativity and creative thinking in contrast to inattention, especially in DT. These symptoms are found in different importance across the presentations of ADHD (APA, 2013), meaning DT abilities and self-rated creativity may vary between these presentations.

Thus, the first objective of this study was to explore creativity as measured by self-report scales and DT tasks in the two most prevalent presentations of ADHD in adults: ADHD-I and ADHD-C (Vitola et al., 2017), in comparison with adults who do not have ADHD. We predicted that adults with ADHD would rate their creativity higher than adults without ADHD and present a superior performance in a DT task. We also predicted that ADHD-I and ADHD-C would differ in these regards since they present different symptoms and possibly varying inhibition fragilities. Adults with ADHD-C may be more creative than adults with ADHD-I since they present more symptoms positively related to higher creativity. Our second objective was to examine whether self-reported creativity and DT were related to ADHD symptoms and executive inhibition, more specificly response inhibition, expecting that superior creativity would be associated with higher levels of ADHD symptoms and poorer executive inhibition.

Aside from the behavioral and cognitive heterogeneity observed in ADHD, some methodological inconsistencies could also account for the variability of the results found in the literature on creativity in ADHD. Medication for ADHD is not consistently controlled, despite evidence that psychostimulants may interact with creativity (Ten et al., 2020; although see Hoogman et al., 2020). Creativity may also be shaped by certain sociodemographic variables. Castillo-Vergara et al. (2018) found that, in fifth-grade students, creative potential increased with social-economic status, and girls exhibited higher creativity than boys in terms of fluency, flexibility, and originality. Thus, we controlled for medication and these sociodemographic variables to the best of our capabilities.

## Methods and Materials

### Participants

Eighty-three participants from the region of Montreal, male (*n* = 20) and female (*n* = 63), within the age of 18 to 51 (mean [*M*] = 26.66, standard deviation [*SD*] = 8.73), took part in the study. The control group counted 43 participants and the clinical groups were composed of participants who had an official diagnosis of ADHD given by a licensed psychiatrist, physician, neuropsychologist, or psychologist. Twenty-one participants were diagnosed with ADHD-I and 19 were diagnosed with ADHD-C. Demographic data of our groups are presented in Table 1. Participants were required not to take their medication for ADHD during test day. Those treated with non-stimulant molecules (e.g., Atomoxetine) were not recruited since their therapeutic effects persist beyond their direct pharmacologic effects (Stahl & Grady, 2017). Other neurodevelopmental disorders such as language disorders, motor disorders, autism spectrum disorder, Tourette syndrome, or those who presented with neurological disorders and moderate or severe traumatic brain injuries also constituted exclusion criteria. However, people with specific learning disorders or anxiety and mood disorders were allowed to participate since these are highly comorbid with ADHD (DuPaul et al., 2013; Katzman et al., 2017). Thus, five participants (control group: *n* = 1, ADHD-C group: *n* = 4) reported having dyslexia. Three participants of the ADHD-C group reported having an anxiety or mood disorder. Our results were not affected by the inclusion of these participants. Finally, a portion of the testing was carried out during the COVID-19 pandemic. Three participants from the ADHD-I group and one from the ADHD-C group were assessed by teleconference. Again, our results were not affected by their inclusion.

**Table 1.** Groups Demographic Data (Age, Sex, and Academic Level).

Group (*n*)	Control (43)	ADHD-I (21)	ADHD-C (19)
Male: *n*, %	13, *30*	5, *24*	2, *11*
Age: *M*(*SD*)	24.60 (*8.35*)	27.67 (*8.25*)	30.21 (*9.18*)
Academic level: <bachelor’s degree	20.9%	42.9%	31.6%
≥Bachelor’s degree	79.1%	57.1%	68.4%

*Note*. ADHD-C = combined presentation of attention-deficit/hyperactivity disorder; ADHD-I = predominantly inattentive presentation of attention-deficit/hyperactivity disorder.

### Procedure

Participants were recruited with ads posted on Facebook and emails sent to students from colleges with whom we had a partnership. Confirmation of eligibility and consent were obtained through a form online. During 2-hour sessions, participants had to complete a self-report creativity scale to measure their perception of their creativity, a formal task of creativity to assess creative abilities, a response inhibition task to measure executive inhibition, and a self-report ADHD questionnaire to measure ADHD symptoms. A nonverbal reasoning task was also administered to verify if differences found between the groups in DT task could not be better explained by differences in nonverbal intelligence. Evaluations were conducted by either one of the four trained investigators under the supervision of an accredited neuropsychologist. Tests were always administered in the same order and were corrected by two scorers who were blind to the group and demographic data.

### Measures

#### Self-reported creativity

The Kaufman Domains of Creativity Scale (K-DOCS) is a 50 items questionnaire assessing self-perceived creative abilities (Kaufman, 2012). Examinees are asked to rate on a scale from 1 to 5 (“much less creative” to “much more creative”) how creative they are compared to their peers. Five domains are evaluated: Self/Everyday (e.g., “Finding something fun to do when I have no money”), Scholarly (e.g., “Debating a controversial topic from my own perspective”), Performance (e.g., “Shooting a fun video to air on YouTube”), Mechanical/Scientific (e.g., “Solving math puzzles”), and Artistic (e.g., “Making a sculpture or piece of pottery”). For these domains, Kaufman (2012) reports internal consistency coefficients of.86,.86,.87,.86, and.83 respectively, as well as two weeks test-retest reliability of .80, .76, .86, .78, and .81. Raw total scores for each domain were used for analysis.

#### DT Task

The figural Torrance Tests of Creative Thinking (TTCT; Torrance, 2008) are three timed drawing activities requiring the production of recognizable images telling a story from different basic stimuli (e.g., a “Y” shape). Images have to be as different as possible from the ones anyone else could do. The main scoring measures are Fluency, Originality, Elaboration, Abstractness of Titles, and Resistance to Premature Closure. Their definitions, as presented in the most recent version of the technical manual (Torrance, 2008), can be found in Table 2. The figural TTCT is reliable and valid (Kim, 2011). Raw scores of each measure were used for analysis.

**Table 2.** Definitions of the Different Measures of the Figural TTCT.

Measure	Definition
Fluency	The number of drawings where the stimulus is used in a meaningful way.
Originality	Statistical infrequency, or “unusualness,” of the response.
Elaboration	Amount of pertinent detail (idea, piece of information, etc.) added to the original stimulus figure, its boundaries, and/or its surrounding space.
Abstractness of titles	Ability to capture the essence of the information involved in the drawings.
Resistance to premature closure	Ability to resist quickly close an incomplete figure with a straight line.

*Note*. TTCT = Torrance Tests of Creative Thinking.

#### Executive inhibition

The Stroop Color and Word Task (Stroop Task; Stroop, 1935) is most often coined as a prepotent response inhibition task (Macleod, 2007). The first three conditions were administered: color naming, word reading, and color-word reading. After naming the colors of a grid of squares that are either blue, green, or red and reading a grid of words that are either “Blue,” “Green,” or “Red,” examinees are presented with a grid of mismatched words and ink color (e.g., the word “Blue” is printed in red). They have to perform a less automated task (naming ink color) while inhibiting automated responses (reading words). Those three conditions of the Color-Word Interference Test from the Delis–Kaplan Executive Functioning System (Delis et al., 2001) were administered, but only the total number of errors and completion time of the color-word reading condition were retained for our analysis. Scaled scores were used to compare groups on executive inhibition and raw scores were used for our main analyses. This subtest presents test-retest reliability of .62 to .76.

#### ADHD symptoms

The self-report version of Conners’ Adult ADHD Rating Scales (CAARS; Conners et al., 1999) is a 4 points Likert-type scale of ADHD symptoms measuring inattention, hyperactivity, and impulsivity symptoms. It has a test-retest reliability of .80 (Conners et al., 1999). *T* scores were used to compare groups on symptoms and raw scores were used for our main analyses.

#### Nonverbal intelligence

The Matrix Reasoning subtest of the Wechsler Adult Intelligence Scale—Fourth Edition (WAIS-IV; Wechsler, 2008) is a sequence of untimed visual puzzles requiring the use of logical and analogical reasoning correlating strongly with the perceptual reasoning index of the WAIS-IV (Wechsler, 2008). Scaled scores were used for verifying group equivalence of nonverbal abilities.

### Data Analysis

Statistical analyses were performed using IBM SPSS Statistics (version 26). In our first objective, groups were compared on K-DOCS domains and measures of the figural TTCT (Fluency, Originality, Elaboration, Abstractness of Titles, and Resistance to Premature Closure) with one-way analyses of variance (ANOVAs). For our second objective, bilateral Pearson’s correlations were used to evaluate if creativity scores in figural TTCT or K-DOCS were related to completion time and the number of errors in the color-word condition of the Stroop Task additionally to the CAARS symptom scales. To limit the number of analyses, correlations were only conducted in groups and in measures where significantly higher creativity scores were found priorly in the results of Objective 1.

## Results

### Preliminary Analyses

Scores were distributed normally in all groups. Homogeneity of variances (*p* > .05) was confirmed by Levene’s test. Tuckey’s correction was used to control for the number of comparisons and the criterion for statistical significance was set at *p* < .05. As indicated by one-way ANOVAs, groups were equivalent on age (*F*[2, 80] = 3.047, *p* = .053, η^2^ = .071) and estimated non-verbal intelligence (*F*[2, 80] = 0.062, *p* = .940, η^2^ = .002); Control group: *M* = 11.62, *SD* = 2.58; ADHD-I group: *M* = 11.38, *SD* = 3.20; ADHD-C: *M* = 11.42, *SD* = 2.32). Chi-squared test showed that groups were equivalent on sex, *X*^2^ (2, *N* = 83) = 2.799, *p* = .247, *V* = 0.184, and academic level, *X*^2^ (2, *N* = 83) = 3.385, *p* = .184. *V* = 0.202.

#### Interrater reliability

The scoring of the figural TTCT is partly subjective, so interclass correlation coefficients (ICCs) were calculated to estimate interrater reliability of its measures on a subset of 20 randomly chosen copies rated at different time points by our two scorers. ICCs estimates and their 95% confidence intervals were calculated with two-way random-effect models based on single ratings and absolute agreement. According to the guidelines of Landis and Koch (1977), the ICCs of Fluency, Originality, Elaboration, and Resistance to Premature Closure were almost perfect being respectively 0.96 (0.91–0.99), 0.92 (0.81–0.97), 0.90 (0.77–0.96), and 0.92 (0.81–0.97). The ICC of Abstractness of Titles was moderate being 0.60 (0.21–0.82). Overall, interrater reliability was adequate.

#### ADHD symptoms and executive inhibition

To verify if the clinical groups presented the ADHD symptoms corresponding to their specific ADHD presentations (APA, 2013) additionally to poorer executive inhibition than controls (Nigg, 2001; Nigg et al., 2005), groups were compared on CAARS and Stroop Task with one-way ANOVAs. There were significant effects of groups on the *T* scores of every CAARS scale (see Table 3). As expected, the pairwise analysis shows that both ADHD groups had a significantly higher Inattention score than the control group (see Table 4). The ADHD-C group also had significantly higher Hyperactivity and Impulsivity scores compared to other groups. To the Stroop Task, there was a large and significant effect of groups on the scaled scores for completion time and number of errors. ADHD-I and ADHD-C participants were significantly slower than controls (see Table 3). Participants with ADHD-I also made more errors than controls (see Table 4).

**Table 3.** One-way ANOVAs Comparing Groups on CAARS Scales (*T* Scores) and Stroop Task (Standard Scores).

Measure	Group	*n*	*M*(*SD*)	dfNum	dfDem	*F*	*p*	η^2^
Inattention (CAARS)	Control	42	49.07 (8.69)					
	ADHD-I	21	63.10 (13.06)	2	78	26.18	.000	.402
	ADHD-C	19	66.53 (7.93)					
Hyperactivity (CAARS)	Control	42	45.07 (8.56)					
	ADHD-I	21	49.29 (9.64)	2	78	17.58	.000	.311
	ADHD-C	19	60.26 (10.19)					
Impulsivity (CAARS)	Control	42	44.71 (7.91)					
	ADHD-I	21	49.05 (11.67)	2	78	15.54	.000	285
	ADHD-C	19	60.11 (11.81)					
Completion time (Stroop Task)	Control	43	11.56 (2.36)					
	ADHD-I	18	8.89 (3.58)	2	77	6.64	.002	.147
	ADHD-C	19	9.32 (3.73)					
Number of errors (Stroop Task)	Control	43	10.84 (1.62)					
	ADHD-I	18	8.33 (3.63)	2	77	7.04	.002	.155
	ADHD-C	19	9.95 (2.39)					

*Note.* ADHD-C = combined presentation of attention-deficit/hyperactivity disorder; ADHD-I = predominantly inattentive presentation of attention-deficit/hyperactivity disorder; CAARS = Conners’ Adult ADHD Rating Scales.

**Table 4.** Pairwise Comparisons of Groups on CAARS Scales (*T* Scores) and Stroop Task (Standard Scores).

Measure	Groups			*MD*	*SE*	*p*
Inattention (CAARS)	Control	—	ADHD-I	−14.02	2.64	.000
	Control	—	ADHD-C	−17.45	2.73	.000
	ADHD-I	—	ADHD-C	−3.43	3.12	.517
Hyperactivity (CAARS)	Control	—	ADHD-I	−4.21	2.48	.212
	Control	—	ADHD-C	−15.19	2.56	.000
	ADHD-I	—	ADHD-C	−10.98	2.93	.000
Impulsivity (CAARS)	Control	—	ADHD-I	−4.34	2.67	.242
	Control	—	ADHD-C	−15.40	2.76	.000
	ADHD-I	—	ADHD-C	−11.06	3.15	.002
Completion time (Stroop Task)	Control	—	ADHD-I	2.67	0.85	.007
	Control	—	ADHD-C	2.24	0.83	.023
	ADHD-I	—	ADHD-C	−0.43	0.90	.903
Number of errors (Stroop Task)	Control	—	ADHD-I	2.50	0.67	.001
	Control	—	ADHD-C	0.89	0.66	.369
	ADHD-I	—	ADHD-C	−1.61	0.78	.105

*Note.* ADHD-C = combined presentation of attention-deficit/hyperactivity disorder; ADHD-I = predominantly inattentive presentation of attention-deficit/hyperactivity disorder; CAARS = Conners’ Adult ADHD Rating Scales.

### Objective 1

One-way ANOVAs revealed a large and significant effect of groups on the K-DOCS Performance domain, as presented in Table 5. Pairwise analysis, shown in Table 6, indicated that the ADHD-C group presented a significantly higher score than both the control and ADHD-I groups on the Performance domain. Other comparisons were not significant. On the figural TTCT, one-way ANOVAs showed significant and large effects of groups in Originality and Abstractness of Titles (Table 5). Pairwise analysis revealed that only participants with ADHD-C displayed a significantly higher score than the controls for these measures (Table 6). No other significant differences were found between groups.

**Table 5.** One-Way ANOVAs Comparing Groups on K-DOCS and Figural TTCT.

Measure	Group	*n*	*M*(*SD*)	dfNum	dfDem	*F*	*p*	η^2^
Self/everyday (K-DOCS)	Control	41	39.32(5.06)	2	78		.090	
	ADHD-I	21	42.23(6.13)			2.48		.060
	ADHD-C	19	41.26(4.07)					
Scholarly (K-DOCS)	Control	41	36.66(6.44)					
	ADHD-I	21	35.62(9.04)	2	78	0.36	.700	.009
	ADHD-C	19	37.42(4.14)					
Performance (K-DOCS)	Control	41	24.00(7.70)					
	ADHD-I	21	26.05(10.40)	2	78	8.49	.000	.179
	ADHD-C	19	34.00(9.18)					
Mechanical/scientific (K-DOCS)	Control	41	21.84(6.04)					
	ADHD-I	21	23.52(7.83)	2	78	1.32	.272	.033
	ADHD-C	19	24.13(7.93)					
Artistic (K-DOCS)	Control	41	30.02(8.98)					
	ADHD-I	21	32.76(7.15)	2	78	1.32	.274	.033
	ADHD-C	19	33.53(9.67)					
Fluency (TTCT)	Control	43	17.54(6.01)					
	ADHD-I	21	19.33(6.81)	2	80	1.98	.144	.047
	ADHD-C	19	21.00(7.13)					
Originality (TTCT)	Control	43	11.16(4.99)					
	ADHD-I	21	14.00(6.01)	2	80	5.41	.006	.119
	ADHD-C	19	16.26(7.29)					
Elaboration (TTCT)	Control	43	12.33(3.62)					
	ADHD-I	21	12.10(3.24)	2	80	0.21	.809	.005
	ADHD-C	19	12.79(3.21)					
Abstractness of titles (TTCT)	Control	43	11.30(5.97)					
	ADHD-I	21	13.48(4.73)	2	80	3.21	.046	.074
	ADHD-C	19	15.47(7.72)					
Resistance to premature closure (TTCT)	Control	43	12.19(4.19)					
	ADHD-I	21	13.29(4.79)	2	80	2.97	.057	.069
	ADHD-C	19	15.05(3.89)					

*Note*. ADHD-C = combined presentation of attention-deficit/hyperactivity disorder; ADHD-I = predominantly inattentive presentation of attention-deficit/hyperactivity disorder; K-DOCS = Kaufman Domains of Creativity Scale; TTC = Torrance Tests of Creative Thinking.

**Table 6.** Pairwise Comparisons of Groups on K-DOCS and Figural TTCT.

Measure	Groups			*MD*	*SE*	*p*
K-DOCS (Performance)	Control	—	ADHD-I	−2.05	2.36	.663
	Control	—	ADHD-C	−10.00	2.45	.000
	ADHD-I	—	ADHD-C	−7.95	2.79	.015
	Control	—	ADHD-I	−2.84	1.55	.168
Figural TTCT (Originality)	Control	—	ADHD-C	−5.10	1.61	.006
	ADHD-I	—	ADHD-C	−2.26	1.85	.442
Figural TTCT (Abstractness of Titles)	Control	—	ADHD-I	−2.17	1.63	.383
	Control	—	ADHD-C	−4.17	1.69	.041
	ADHD-I	—	ADHD-C	−2.00	1.94	.562

*Note.* ADHD-C = combined presentation of attention-deficit/hyperactivity disorder; ADHD-I = predominantly inattentive presentation of attention-deficit/hyperactivity disorder; K-DOCS = Kaufman Domains of Creativity Scale; TTCT = Torrance Tests of Creative Thinking.

### Objective 2

Bilateral Pearson’s correlations presented in Table 7 showed that the total raw score for the hyperactivity scale of the CAARS was moderately positively correlated to the score in the performance domain of the K-DOCS in participants with ADHD-C. No significant correlation was found between figural TTCT measures and CAARS scales or Stroop Task’s measures.

**Table 7.** Bilateral Pearson’s Correlations of Stroop Task, K-DOCS, and Figural TTCT in ADHD-C group.

Measure		Stroop Task		CAARS (total raw score)		
		Time (s)	Errors	Inattention	Hyperactivity	Impulsivity
Performance (K-DOCS)	*r*	.390	.252	.00	.575	.420
	*p*	.099	.299	1.00	.010	.074
	*N*	19	19	19	19	19
Originality (Figural TTCT)	*r*	−.025	−.340	.416	−.025	−.219
	*p*	.919	.155	.076	.919	.368
	*N*	19	19	19	19	19
Abstractness of titles (Figural TTCT)	*r*	−.230	−.242	.289	−.080	.191
	*p*	.343	.319	.230	.744	.433
	*N*	19	19	19	19	19

*Note*. ADHD-C = combined presentation of attention-deficit/hyperactivity disorder; ADHD-I = predominantly inattentive presentation of attention-deficit/hyperactivity disorder; CAARS = Conners’ Adult ADHD Rating Scales; K-DOCS = The Kaufman Domains of Creativity Scale; TTCT = Torrance Tests of Creative Thinking.

## Discussion

In this study we investigated creativity in adults with or without ADHD, our main contribution being to explicitly distinguish between two ADHD presentations (ADHD-I and ADHD-C). We also verified if superior creativity between groups was related to higher ADHD symptoms and lower executive inhibition. Our results provide evidence that creativity varies between ADHD presentations, and we propose it might be mainly explained by differences in behavioral symptoms.

Our first finding was that participants with ADHD-C self-rated their creativity higher than the other groups on the K-DOCS, more precisely on the Performance domain, which encompassed music, theater, and dance. Boot et al. (2017a) also found higher self-rated creativity scores in adults with ADHD compared to controls in the Performance domain, but also in the Mechanical/Scientific domain. This disparity either stands from a lack of statistical power on our behalf or differences between our clinical samples, as the authors did not control for ADHD presentations (Boot et al., 2017a). Regarding other creativity scales, higher self-rated creativity in adults with ADHD was also found in case-control studies that measured the number of real-world creative achievements (Boot et al., 2017a; DuPaul et al., 2017; White & Shah, 2011), but the different domains of creativity were not considered.

The score in the Performance domain of the ADHD-C group was moderately and positively related to hyperactivity and marginally, but not significantly, related to impulsivity (*r* = .420, *p* = .074). Interestingly, these symptoms are linked to extraversion (Krieger et al., 2020), a personality trait found in high levels in singers, comedians, and actors (Cameron et al., 2015; Greengross & Miller, 2009; Nettle, 2006). Extraversion would ease performers into taking the center stage, among other things. Thus, hyperactivity and impulsivity could, similarly to extraversion, make individuals with ADHD-C feeling comfortable and possibly even motivated to do acts related to the performance domain. To our knowledge, the present research was the first to examine the relation between the different symptoms of ADHD and self-rated creativity within a clinical sample. However, a study in adults without ADHD revealed that more self-reported impulsivity and hyperactivity symptoms were correlated to more creative achievements, but again, in unspecified domains (Boot et al., 2017b). Therefore, there seems to be a link between self-reported hyperactivity and impulsivity and self-rated creativity, but its specificity to certain domains needs to be further studied. Moreover, since these domains often encompass heterogenous subdomains of creativity (e.g., the Performance domain in K-DOCS covers acts of writing and playing music instruments), an item analysis would help to identify more precisely the kind of activities in which individuals with ADHD feel they are particularly creative.

Our second important and novel finding was that participants with ADHD-C also produced more original drawings paired to more abstract titles, but only compared to the controls. This is in line with the tendency to detect higher originality scores in adult participants with ADHD compared to controls in a variety of DT measures, as reported in the systematic review of Hoogman et al. (2020). With regards to Abstractness of Titles, Fugate et al. (2013) have observed the same outcome in gifted children with ADHD compared to their peers without the disorder.

More self-reported hyperactive and impulsive symptoms were not linearly associated with better performances to the DT task in participants with ADHD-C, contrarily to the findings of other studies in participants without ADHD (Boot et al., 2017b; Brandau et al., 2007). In ADHD, this relation may be rather curvilinear. Up to a certain point, more ADHD symptoms could be associated with more cognitive impairments, which ultimately lowers DT performances, similarly to schizotypy symptoms and DT (Acar & Sen, 2013). While Boot et al. (2017b) did not found such a curvilinear relation and did not found more cognitive deficits in participants self-reporting more ADHD symptoms, the number of participants with clinically elevated symptoms of ADHD within their sample (17%) was likely overestimated if compared to the prevalence of ADHD in the general population (2.58%; Song et al., 2021). A clinical sample of participants officially diagnosed with ADHD may offer a better representation of the gradient of cognitive deficits associated with higher levels of ADHD symptoms. Interestingly, there was a moderate and positive, but not significant, relationship between the Originality score and the number of self-reported inattentive symptoms (*r* = .416, *p* = .076). Perhaps, curvilinear analyses would be more adequate for measuring the relation between the behavioral symptoms of ADHD and creativity.

The ADHD-I group was not creatively superior to the other groups. This is coherent with previous works suggesting that the main drivers of ADHD are hyperactivity/impulsivity (Boot et al., 2017b; Brandau et al., 2007), two symptoms self-reported in smaller importance in ADHD-I. Nonetheless, mind wandering (an inattention-like behavior) should enhance creativity in DT (Baird et al., 2012), but this typically implies an incubation period that occurs while taking a break from the task at hand. Moreover, in adults without ADHD, a more flexible attention allowing rapid changes in modes of focus is associated with higher performances to DT tasks, which requires quick transitions between ideas (Zabelina et al., 2016). Conversely, a “leaky” attention that would not properly filter out irrelevant information, which shares more likeness to the attentional profile found in ADHD, has been associated with more creative achievements, but not DT. Thus, the qualities of the DT task chosen, a timed-constrained activity that did not allow pauses for mind wandering and that may have benefited from a flexible attention rather than inattentiveness could have been disadvantageous for participants with ADHD-I.

Finally, there is little evidence in our results supporting our hypothesis that higher DT scores would be related to poorer executive inhibition. In ADHD-C, poorer executive inhibition was marginally and positively, but not significantly, related to a higher score in the Performance domain (*r* = .390, *p* = .099), which echoes the findings of Carson et al. (2003) who reported higher self-rated creativity in adults with poorer latent inhibition. Executive inhibition appeared to be most impacted in participants with ADHD-I, compared to controls. Yet, these groups were not creatively different. Thus, our results do not strongly support either hypothesis of a positive contribution of higher and poorer executive inhibition to creativity. They are rather in line with other works that found no clear association between these two concepts (Burch et al., 2006; Green & Williams, 1999; Stavridou & Furnham, 1996). It is worth noting that prepotent response inhibition is only a facet of response inhibition, and more largely of inhibition functioning. This has only been little studied in ADHD, so further investigation is needed to determine if poorer executive inhibition has any positive implications on creativity in this population.

An important limitation of this study was the sample size. Because our recruitment was impacted by the COVID-19 pandemic, we were not able to obtain the sample size initially planned for our clinical groups. More statistical power could confirm (or disconfirm) the tendencies we underlined. We particularly had difficulties recruiting male participants, especially in our clinical groups. Moreover, our groups were also almost nonequivalent regarding age. Fortunately, most studies report no gender-based differences in creativity (Abraham, 2016). While aging has been shown to fragilize inhibitory mechanisms and consequently, facilitate DT, this has been observed in a much older sample (aged 60–77; Carpenter et al., 2020). Thus, it is unlikely that these variables had a significant effect on our results.

## Conclusion

At the time of writing, this was the first study that compared creativity between clinical samples of individuals officially diagnosed with ADHD-I and ADHD-C. Taken together, our results suggest there are variations of creativity between these two presentations, with higher creativity scores exclusively being found in ADHD-C. These higher scores appeared to be linked more importantly to behavioral symptoms than poorer executive inhibition. Our results are novel and have several important implications for future research on ADHD and creativity. They highlight the importance of considering the presentations of ADHD in research on creativity. ADHD presentations are associated with different sets of behavioral symptoms, and some appear to contribute better than others to certain facets of creativity. This is not to say individuals with ADHD-I are not highly creative. The domain or in which they exude creativity might simply be unknown yet. Moreover, if mind-wandering and inattentiveness are the entryways of creativity in ADHD-I, time-constrained tasks may not be the best suited for measuring this ability in this population. Future work should recognize the heterogeneity of ADHD but also aim to refine the identification of the domains and the contexts in which individuals with different presentations of ADHD may excel creatively. It will then be possible to better orient this group toward success.
